# Virucidal gargling and virucidal nasal spray 

**DOI:** 10.3205/dgkh000373

**Published:** 2021-01-18

**Authors:** Axel Kramer, Maren Eggers, Nils-Olaf Hübner, Peter Walger, Eike Steinmann, Martin Exner

**Affiliations:** 1 Institute of Hygiene and Environmental Medicine, University Medicine Greifswald, Germany; 2 Labor Prof. Gisela Enders MVZ GbR, Stuttgart, Germany; 3 Central Unit for Infection Prevention and Control, University Medicine Greifswald, Greifswald, Germany; 4 German Society of Hospital Hygiene, Berlin, Germany; 5 Internal Intensive Medicine and Infectiology, Evangelic Clinics Bonn, Johanniter-Krankenhaus, Bonn, Germany; 6 Institute of Hygiene and Microbiology, Department for Molecular & Medical Virology, Ruhr-University Bochum, Bochum, Germany; 7 Institute for Hygiene and Public Health, University Hospital Bonn, Bonn, Germany

## Introduction

To prevent COVID-19, all available hygienic measures must be implemented, especially to protect the medical staff, but also the community. Since a large proportion of those infected release the virus before initial symptoms appear, protective measures that reduce the viral load at the entry points are useful, since the probability of infection increases with exposure, and the initial viral load influences the severity of the infection [1], [2].

Antiseptic gargling and nasal antiseptics are simple preventive measures that have needlessly been forgotten. Gargling has long been used to reduce upper respiratory tract infections and to treat bacterial/viral infections (e.g. sore throats, colds), but it has gone out of fashion. Washing hands with soap and water and gargling with saline solution was already recommended as a preventive measure by the German Health Council during the Spanish flu epidemic in 1918 [3]. In the former GDR, school children were advised to gargle with diluted potassium permanganate solution (pale pink) when entering summer holiday camp (author’s experience). Potassium permanganate solution is hardly used any more today, although it is available as a 1% mouth rinse solution.

Unlike in Europe, daily gargling has a long tradition in Japan for the prevention of respiratory infections, and was strongly promoted by the Japanese Ministry of Health, Labour and Welfare during the H1N1 swine flu pandemic in 2009. It has now been explicitly recommended again to the population for daily use in the COVID-19 pandemic.

It has been known for centuries that salty air has a beneficial, soothing effect on the respiratory tract. It stimulates the natural self-cleaning of the respiratory tract and prevents the mucous membranes from drying out. In addition, moistening the mucous membranes of the mouth and nose reduces the adhesion of viruses and therefore has a preventive effect even without the use of solutions or sprays with a virucidal effect [4], [5].

To use gargling and nasal rinsing as easily implementable measures for the prevention and control of COVID-19 in Germany, a short summary of previous knowledge on the virucidal effect and the preventive uses of gargling solutions and nasal sprays is given to exploit a further reserve of prevention and promote the public-health discussion process. The advent of the COVID-19 pandemic has made social distancing, mouth and nose protection, hand disinfection and ventilation of indoor spaces vitally important for prevention within the population. Similarly, these easy preventive measures should also be used more intensively.

## Current knowledge on the virucidal effectiveness of gargles and nasal spray

If no source is cited for the following statements, the cited overview [6] contains the references.

### In vitro efficacy

Efficacy against SARS-CoV-2 has been demonstrated *in vitro* for the following formulations: nasal spray based on Carragellose [7], [8] and PVP iodine ≥0.23%, mouthwashes based on essential oils, dequalinium chloride and benzalkonium chloride (Dequonal), phenoxyethanol + octenidine (Octenisept) [9], [10], ethanol + ethyl lauryl arginate and two formulations based on cetyl pyridinium chloride [11]. For mouthwashes based on essential oils, complete inactivation of SARS-CoV-2 was demonstrated with alcohol content (Listerine Cool Mint) as well as without (Listerine Cool Mint mild taste) [12]. In contrast, mouthwashes based on hydrogen peroxide, polihexanide, chlorhexidine or octenidine (the latter without the combination with phenoxyethanol) were not sufficiently effective [10].

Green tea, pomegranate and aronia juice are also virucidal, but they are not as effective as the above-mentioned active ingredients or their combinations. After 1 minute of exposure to green tea and pomegranate juice, the infectiousness of the viruses is reduced by 80% and by 97% with aronia juice [13].

Sage extract has been shown to be effective against influenza and other corona viruses, so there is a high probability that it is also effective against SARS-CoV-2.

### Effectiveness in different trials

**Gargling with preventive intention**

- With hypertonic saline solution (2%–3%) 3 times/d: significantly shortened the infection in the user; by reducing the excretion of the virus, the disease incidence was also significantly reduced by 35% in persons living in the same household.
- With green tea: reduction of viral influenza diseases by 30% (comparison with water or no gargling; 5 studies).
- With PVP iodine 7%: significantly fewer days of school failure due to colds and flu.

**Gargling with therapeutic intention**

- In patients in stage 1 of COVID-19 (=pre-symptomatic stage 1–2 days before onset of symptoms after infection [14]), viral clearance was significantly increased by both 1% PVP iodine and the combination of ethanol with essential oils compared to tap water [15]. In a small case study in Spain, 1% PVP iodine also reduced the viral load in COVID-19 patients [16].

**Nasal spray**

- Carragelose (Algovir^®^ cold spray: 1.2 mg carragelose +0.5% NaCl): significantly shortened the number of patients and the duration of illness (3 studies [17]).

**Mouth rinsing**

- Sage extract: as effective against Herpes labialis as the virostatic drug Aciclovir.
- Combination of ethanol with essential oils: similarly highly effective against Herpes labialis.

## Risk assessment for long-term use

- Carragelose (red algae extract), ethanol + essential oils, saline solution and green tea: no risks.
- 0.23% PVP iodine: when applied on eyes, max. 1.8% of the applied iodine is absorbed. The absorption during gargling has not been studied. Under the worst-case assumption of 10% absorption, 1.000 µg of iodine would be absorbed with one gargle, which is 5 times the WHO-recommended daily iodine intake through food. In the context of topical applications, iodine-induced hyper- or hypothyroidism is only described in the case of excess exposures that are many times higher than those possible with oral gargling. Single cases of thyroid dysfunction have been reported for urinary bladder or peritoneal irrigation or for irrigation of large wounds [18], [19], [20], [21]. Frank et al. [22] conclude that the use of PVP-I in the oral cavity in concentrations of up to 2.5% is safe for up to 5 months.
- Octenidine: because of pronounced cytotoxicity and irritation potential [23], long-term use could have side effects (mucositis; if absorbed into the lungs at trace levels, there is a possible risk of initially unnoticeable long-term side effects).

## National and international recommendations

### Protection of the population as long as regional clusters and/or 7-day incidence >50

- **Japan:** gargle in the morning and evening and nasal spray with 0.23% aqueous PVP-iodine solution.

### Pre-exposure prophylaxis to protect healthcare workers

- **Belgium**: gargling with 1% PVP iodine.
- **Portugal and Malt**a: gargling with 0.2% PVP iodine.
- **World Health Organization** [24]: gargling with 0.2% PVP iodine.
- **Germany**: gargling with 0.2% PVP iodine before dental treatment [25]; before intubation and bronchoscopy, irrigation of the oral cavity with 1.25% aqueous PVP iodine solution, preferably in combination with gargling [26].

## Recommendations aimed at Germany

### For the community

**A) Gargling **

- **Table salt**: dissolve one level teaspoon of table salt in 100 ml of lukewarm water. Put about a shot glass (glass of brandy) in your mouth, interrupt gargling before inhalation and repeat the process for about 3 minutes; at least 3 times/d in the morning and evening, if possible; do not swallow the gargle solution.
- **Green tea**: cool lukewarm to gargle.
- **Pomegranate and aronia juice**: since only *in vitro* results are available so far, saline solution and green tea are to be preferred.
- **Essential oils**: mouthwashes used undiluted. Mouthwashes based on essential oils are preferable to the above options because of its high *in vitro* effectiveness against SARS-CoV-2 in terms of reducing the viral load in people infected with SARS-CoV-2. There are no known long-term side effects. For children, people with alcohol intolerance and people with particular mucous membrane sensitivity, the formulation without alcohol (Listerine Cool Mint mild flavor) should be used instead of the combination of essential oils with alcohol (Listerine Cool Mint).

**B) Nasal spray **

- **Table salt**: products without the addition of preservatives or decongestants (e.g., Hysan^®^ Salinspray^®^ or Rinupret^®^) or home-made (see above); application: absorb into the nose by inhalation.
- **Carragelose**: (Algovir^®^ Cold Spray) to be preferred because of its higher effectiveness compared to table salt.

### Pre-exposure prophylaxis to protect healthcare workers

Before aerosol-generating measures (e.g., dental treatment, ENT treatment, intubation, non-invasive ventilation): have the patient gargle with 1.25% aqueous PVP-iodine solution (at this concentration, it is also tolerated by the sensitive nasal mucosa). In case of contraindication against iodine (known hyperthyroidism or iodine allergy), instead choose mouthwashes based on essential oils.

Since March 2020, pre-exposure prophylaxis has been carried out at Greifswald University Medicine with 1.25% aqueous PVP iodine solution and, in case of contraindication, with the combination ethanol/essential oils. Since then, there has not been one case of intolerance and no transmission from patients to the physician or dentist.

### Pre-exposure prophylaxis to protect the community

Special emphasis is given to the application, e.g., before taking meals together or before communal activities in institutions for the elderly or in rehabilitation facilities, at family gatherings (in the permitted sizes), at professional group meetings, church services, other religious celebrations, funerals etc.

For employees in health care facilities, it makes sense to first gargle at home and a second time in the facility, to inactivate any viruses that may have adhered while travelling. If infection control nurses are on site in the hospital, the practical implementation of gargling should be discussed and determined with them, so that the environment around the basin is not contaminated when the gargle solution is spit out. In any case, after gargling, the basin should be rinsed with water and then wiped with a cloth soaked in disinfectant solution.

**Recommendation in descending order: **Gargle with mouthwashes based on essential oils, 1.25% PVP iodine (as a spray for dementia), and green tea or saline solution.

When PVP-iodine-based mouthrinses and nasal sprays were tested, PVP-iodine was reliably inactivated at ≥1% SARS-CoV-2 [27]. Since a 1.25% PVP-iodine solution can be produced in any pharmacy in accordance with NRF 15.13 [28], filling in spray bottles is possible as long as no commercial product is available in Germany.

In schools and kindergartens, it is recommended that children and caregivers gargle with green tea or saline solution while simultaneously using nasal spray. Algovir^®^ Cold Spray is the preferred choice, as it is more effective due to its carragelose content. If saline-based nasal sprays without carragelose are selected, it is important to ensure that they do not contain preservatives or decongestants, due to the risk of habituation and chronic mucous membrane damage.

### Post-exposure prophylaxis to protect community healthcare workers as well as the community

**After contact with SARS-CoV-2 positive carriers**: For 7 (to 14) days, gargle and use nasal spray with 0.23% aqueous PVP iodine solution; contraindications are known thyroid diseases and allergy. As neither product is available in Germany, the following options are possible: dilution of Betaisodona mouth antiseptic (3 ml to 100 ml water or pharmacy production of 0.23% aqueous PVP iodine solution); alternatively, nasal spray with carragelose and gargling with ethanol + essential oils.

## Notes

### Competing interests

The authors declare that they have no competing interests.
